# Common Risk Variants in *AHI1* Are Associated With Childhood Steroid Sensitive Nephrotic Syndrome

**DOI:** 10.1016/j.ekir.2023.05.018

**Published:** 2023-05-27

**Authors:** Mallory L. Downie, Sanjana Gupta, Catalin Voinescu, Adam P. Levine, Omid Sadeghi-Alavijeh, Stephanie Dufek-Kamperis, Jingjing Cao, Martin Christian, Jameela A. Kari, Shenal Thalgahagoda, Randula Ranawaka, Asiri Abeyagunawardena, Rasheed Gbadegesin, Rulan Parekh, Robert Kleta, Detlef Bockenhauer, Horia C. Stanescu, Daniel P. Gale

**Affiliations:** 1 Department of Renal Medicine, University College London, London, UK; 2 Paediatric Nephrology, Great Ormond Street Hospital for Children NHS Foundation Trust, London, UK; 3 Department of Pathology, University College London, London, UK; 4 Department of Pediatrics and Adolescent Medicine, Aarhus University Hospital, Aarhus, Denmark; 5 Division of Nephrology, Department of Pediatrics, The Hospital for Sick Children, Toronto, Canada; 6 Nottingham Children’s Hospital, Nottingham, UK; 7 Pediatric Nephrology Centre of Excellence, King Abdulaziz University, Jeddah, Kingdom of Saudi Arabia; 8 Department of Pediatrics, University of Peradeniya, Peradeniya, Sri Lanka; 9 Department of Pediatrics, Duke University School of Medicine, Durham, North Carolina, USA; 10 Department of Medicine, Women’s College Hospital, Toronto, Canada

**Keywords:** AHI1, GWAS, HLA, pediatric nephrology, SSNS

## Abstract

**Introduction:**

Steroid-sensitive nephrotic syndrome (SSNS) is the most common form of kidney disease in children worldwide. Genome-wide association studies (GWAS) have demonstrated the association of SSNS with genetic variation at *HLA-DQ/DR* and have identified several non-*HLA* loci that aid in further understanding of disease pathophysiology. We sought to identify additional genetic loci associated with SSNS in children of Sri Lankan and European ancestry.

**Methods:**

We conducted a GWAS in a cohort of Sri Lankan individuals comprising 420 pediatric patients with SSNS and 2339 genetic ancestry matched controls obtained from the UK Biobank. We then performed a transethnic meta-analysis with a previously reported European cohort of 422 pediatric patients and 5642 controls.

**Results:**

Our GWAS confirmed the previously reported association of SSNS with *HLA-DR/DQ* (rs9271602, *P* = 1.12 × 10^−27^, odds ratio [OR] = 2.75). Transethnic meta-analysis replicated these findings and identified a novel association at *AHI1* (rs2746432, *P* = 2.79 × 10^−8^, OR = 1.37), which was also replicated in an independent South Asian cohort. *AHI1* is implicated in ciliary protein transport and immune dysregulation, with rare variation in this gene contributing to Joubert syndrome type 3.

**Conclusions:**

Common variation in *AHI1* confers risk of the development of SSNS in both Sri Lankan and European populations. The association with common variation in *AHI1* further supports the role of immune dysregulation in the pathogenesis of SSNS and demonstrates that variation across the allele frequency spectrum in a gene can contribute to disparate monogenic and polygenic diseases.

## Graphical abstract

**Figure:**
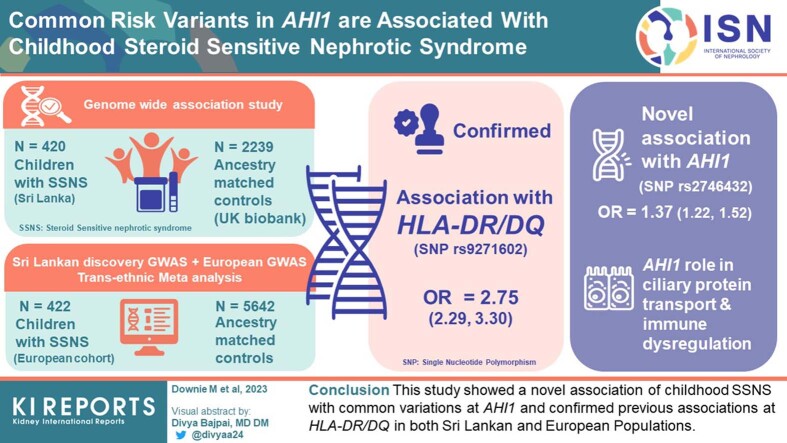

SSNS is the most common kidney disease in children worldwide, with an incidence of approximately 1 to 10 per 100,000.^1^ Incidence varies with ancestry; individuals of South Asian ancestry demonstrate higher risk for the disease than Europeans.^2^ These observations suggest genetic and/or environmental influences in the development of SSNS. Although there have been several studies that have identified genetic risk loci in children with SSNS of European and other ancestries,^3^, ^4^, ^5^, ^6^ additional genetic risk factors associated with SSNS in children of South Asian ancestry have not been reported.

SSNS is characterized by the leakage of protein from the blood into the urine through damaged glomeruli.^7^ The etiology of SSNS remains unclear, though clinical observations suggest an underlying immunologic basis to disease. First, SSNS is defined by response to initial treatment with corticosteroid therapy and in patients that develop a relapsing course of disease, SSNS also responds to additional immunosuppressive medications.^8^ Second, the onset of disease is typically associated with a preceding infection, suggesting that prior activation of the immune system may trigger the disease.^9^ Third, antibodies directed toward nephrin, a protein in the slit diaphragm in the glomerulus, have recently been identified in patients with SSNS.^10^ These clinical observations suggest that SSNS is an autoimmune disorder, implicating both genetic and environmental factors contributing to development of the disease.

GWAS have been instrumental in elucidating genetic risk factors for developing SSNS in childhood. The *HLA-DR/DQ* region has exhibited the strongest association with disease in European, South Asian, and Japanese populations,^3^, ^4^, ^5^, ^6^^,^^11^ supporting the inference from clinical observations that SSNS has an immunologic basis. Beyond the *HLA* region, genome-wide associations at *CALHM6* and *PARM1* have been identified in European children,^3^ and at *NPHS1* and *TNFSF15* in Japanese children.^6^ In the latter study, the *NPHS1* and *TNFSF15* loci were not replicated in a European population, suggesting that SSNS possesses different genetic architecture outside of *HLA* in these 2 different groups. We set out to perform a GWAS in a Sri Lankan population, followed by a European-Sri Lankan transethnic meta-analysis, to identify additional genetic loci associated with SSNS to aid in further understanding of the pathophysiology of disease.

## Methods

Abbreviated methods follow. Detailed methods may be found in the Supplementary Material.

### Study Populations

Sri Lankan patients diagnosed with childhood SSNS (age of onset <18 years) were recruited into the study. Most patients were of self-reported Sri Lankan ancestry, with additional ancestrally matched patients identified by principal component analysis. All patients were diagnosed with SSNS as per the Kidney Disease: Improving Global Outcomes guidelines.^12^ Patients were recruited by collaborating clinicians at their affiliated institutions, as well as from the Prednisolone in Nephrotic Syndrome (PREDNOS, EudraCT 2010-022489-29) and PREDNOS2 (EudraCT 2012-003476-39) trials (Cattran *et al.*^13^ and para 2 of Webb *et al.*^14^). Informed written consent was obtained from each participant and ethical approval was granted at each contributing institution. Ancestrally matched controls were obtained from the UK Biobank.^15^

### Genotyping, Quality Control, and Whole-Genome Imputation

**Figure 1:**
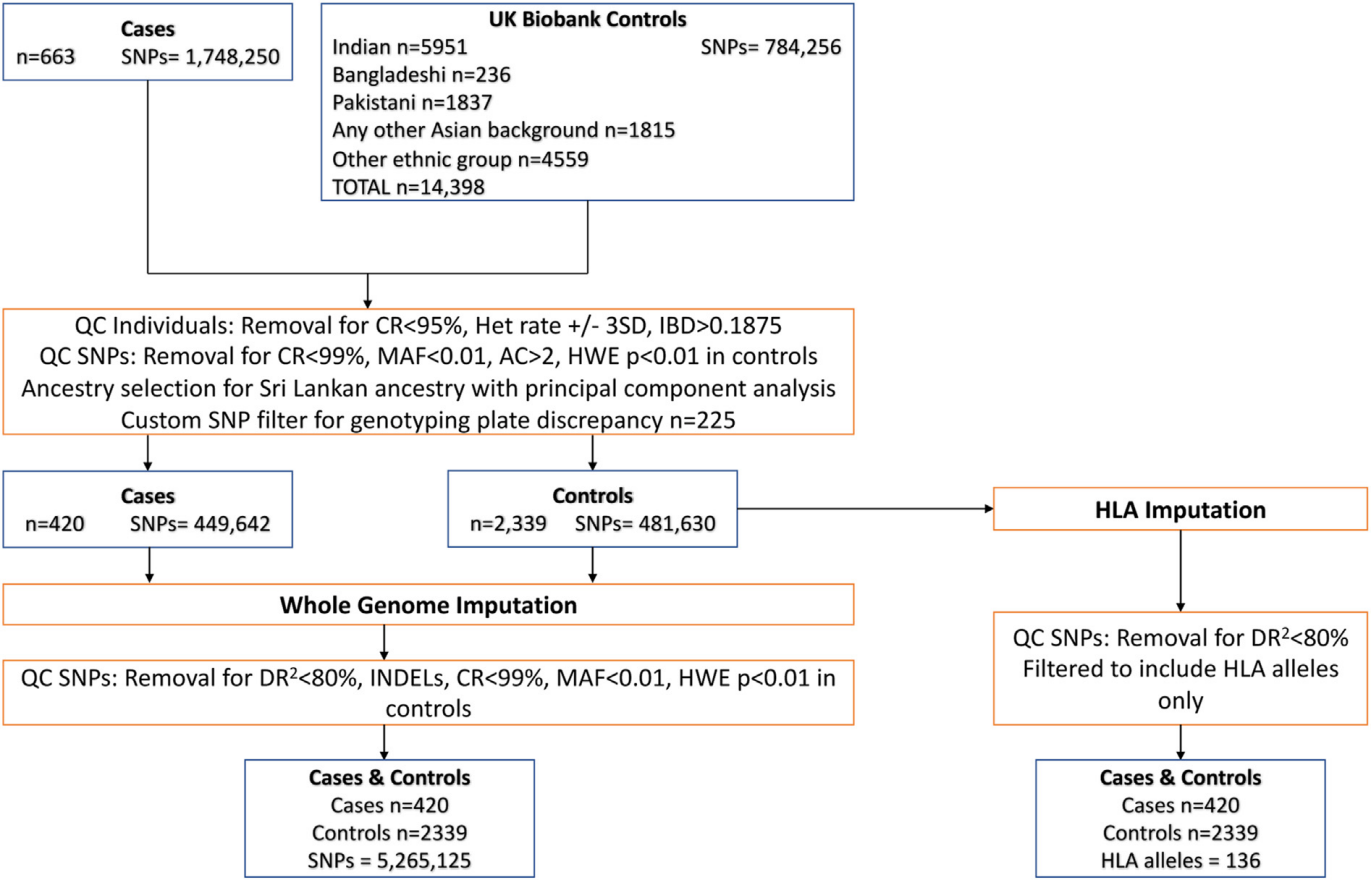
Flowchart of the data processing for the SSNS Sri Lankan discovery GWAS. CR, call rate; Het, heterozygosity; HLA, human leukocyte antigen; HWE, Hardy-Weinberg equilibrium; IBD, identity by descent; INDEL, insertions/deletions; MAF, minor allele frequency; QC, quality control; SNP, single nucleotide polymorphism*;* GWAS*, g*enome-wide association study.

Isolation of DNA and genotyping were performed using standard procedures (see Supplementary Methods). Patients were genotyped via the Infinium Multi-Ethnic Global Array BeadChip v.A1 at University College London Genomics (Institute of Child Health, University College London, UK). UK Biobank controls had been genotyped using the Applied Biosystems UK Biobank Axiom Array. Before imputation, quality control was performed on the case and control cohorts separately (Figure 1). Individuals were excluded by low call rate (<95%), low genotyping quality (heterozygosity rates >3 SDs ± from the mean), and relatedness (IBD ≤ 0.1875). Single-nucleotide polymorphisms (SNPs) were excluded by >2 alleles, low call rate (<99%), low minor allele frequency (<0.01), and in the control cohort only, deviation from Hardy-Weinberg equilibrium (*P* < 0.01). A further filter was applied to both cohorts to remove SNPs genotyped discrepantly between the Multi-Ethnic Global Array BeadChip and Axiom arrays. These SNPs were identified by comparison of a separate group of Sri Lankan healthy control subjects genotyped on the Multi-Ethnic Global Array BeadChip and the control cohort genotyped on the Axiom array (Supplementary Figure S1). Principal component analysis was used to identify the subset of cases and controls of Sri Lankan ancestry (Supplementary Figure S2).

Whole-genome imputation was performed with minimac4 on the Michigan Imputation Server^16^^,^^17^ using the 1000 Genomes Project Phase 3 as the reference panel.^18^ SNPs with a dosage R^2^ of <0.8 were excluded. Postimputation quality control excluded SNPs by low call rate (<99%), low minor allele frequency (<0.01) and deviation from Hardy-Weinberg equilibrium (*P* < 0.01) in controls. PLINK versions 1.90 and 2.00 were used for quality control analysis.^19^

### Genome-Wide Association Analysis

GWAS was performed in SAIGE^20^ with adjustment for sex and the first 3 principal components of ancestry. Using >3 principal components resulted in genomic deflation, suggesting overfitting. Conditional analysis of the lead SNPs was performed in SAIGE using the same model adjusted for sex and principal components. A genome-wide significance threshold of *P* < 5 × 10^−8^ was used. R v4.2.1 was used to generate Manhattan plots. Regional plots were generated using LocusZoom with 1000 Genomes Nov 2014 used as the linkage disequilibrium (LD) reference.^21^

## HLA Fine-Mapping

HLA imputation was performed with minimac4 on the Michigan Imputation Server using the HLA-TAPAS (HLA-Typing at Protein for Association Studies) reference panel.^16^^,^^17^^,^^22^ HLA association analysis was performed in PLINK v2.00 using a logistic regression model adjusted for sex and the first 10 principal components of ancestry. Conditional analysis of the lead HLA allele was performed using the same logistic regression model adjusted for sex and principal components. Association testing and conditional analyses were performed on the HLA 4-digit classical alleles and HLA amino acid polymorphisms separately. Significance thresholds of *P* < 3.0 × 10^−4^ (0.05/136) and *P* < 2.8 × 10^−5^ (0.05/1778) were used to adjust for multiple comparisons with the *n =* 136 4-digit HLA classical alleles and *n =* 1778 HLA amino acid polymorphisms used in the analysis, respectively.

### Transethnic meta-analysis

Transethnic meta-analysis was performed with a previously reported GWAS of European children with SSNS.^3^ Analysis was conducted using the set of overlapping markers between the 2 data sets. The inverse-variance method was used based on a fixed-effects model in META: https://mathgen.stats.ox.ac.uk/genetics_software/meta/meta.html. The genomic inflation factor (λ) and population sizes of each study were corrected for in the model. Results were considered significantly heterogeneous with a Cochran Q test *P* < 0.10. The genome-wide significance threshold for the meta-analysis was considered for *P* < 5 × 10^−8^.

### Replication

Replication of the 2 novel candidate SNPs (in *TMEM131L* and *AHI1*) was assessed in an independent population that comprised 150 South Asian (including Sri Lankan) participants from the INSIGHT^23^ cohort and 277 controls from the Spit for Science study.^24^ South Asian genetic ancestry was determined by principal component analysis using 1000 Genomes^18^ ancestry controls as reference. Association analyses were carried out under an additive model. Significance threshold for replication was considered as *P* < 0.05/2 = 0.025. For replication of candidate SNPs at the *HLA* locus, we examined the results of previously published GWAS in SSNS.^3^, ^4^, ^5^, ^6^

### Power Calculation

The GWAS and replication study power were calculated using the Michigan Genetic Association Study power calculator^25^ assuming a disease prevalence of 1:10,000. For the initial GWAS (420 cases and 2339 controls), the minimum genotype relative risk with a power of 0.8 was calculated using an additive model assuming a disease allele frequency of 0.10 in the control population and a significance level of 5 × 10^−8^. For the replication analysis (150 cases and 277 controls), power calculation assumed the genotype relative risk and allele frequency at each locus observed in the discovery GWAS, with a significance threshold of *P* < 0.025.

## Results

### GWAS Study Cohort

A total of 663 individuals with childhood-onset SSNS and South Asian ancestry were available for our study, and 420 Sri Lankan cases were included in the association analysis following quality control and selection for Sri Lankan ancestry (Figure 1). The control data set was obtained from the UK Biobank from cohorts of self-reported Indian, Bangladeshi, Pakistani, Any other Asian background, and other ethnic group ancestry for an initial total of 14,398 individuals. After ancestrally matching these individuals to our cases and performing quality control, 2339 healthy individuals with genetically determined Sri Lankan ancestry were included in the association analysis, with the majority obtained from the Any other Asian background and other ethnic group cohorts. The case and control cohorts were imputed and combined to yield a total of 5,265,125 high quality SNPs for analysis.

### GWAS Results

**Figure 2:**
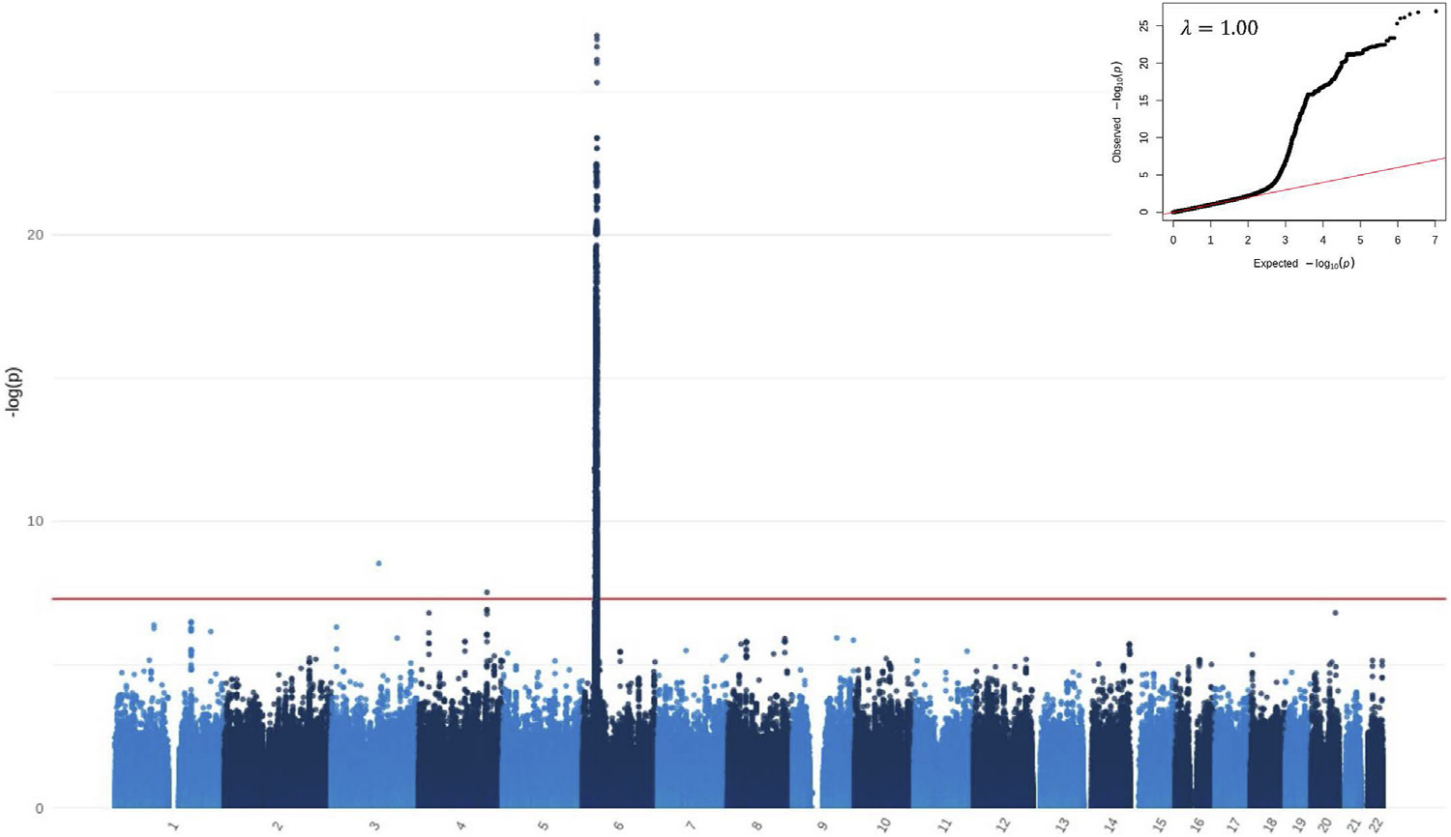
Manhattan plot in Sri Lankan SSNS. GWAS for SSNS in 420 Sri Lankan patients and 2339 ancestrally matched controls. Mixed model logistic regression analysis adjusted for the first 3 principal components was performed in SAIGE. Autosomal chromosomes (1–22) are listed along the x-axis. The level of significance is depicted along the y-axis as −log_10_(P). Each dot represents a variant. The red line represents the threshold of genome-wide significance (P = 5 × 10^−8^). Three loci achieve genome-wide significance on chromosomes 3, 4, and 6. QQ-plot and lambda are displayed in the top right corner. GWAS, Genome-wide association study; SSNS, sensitive nephrotic syndrome.

**Figure 3:**
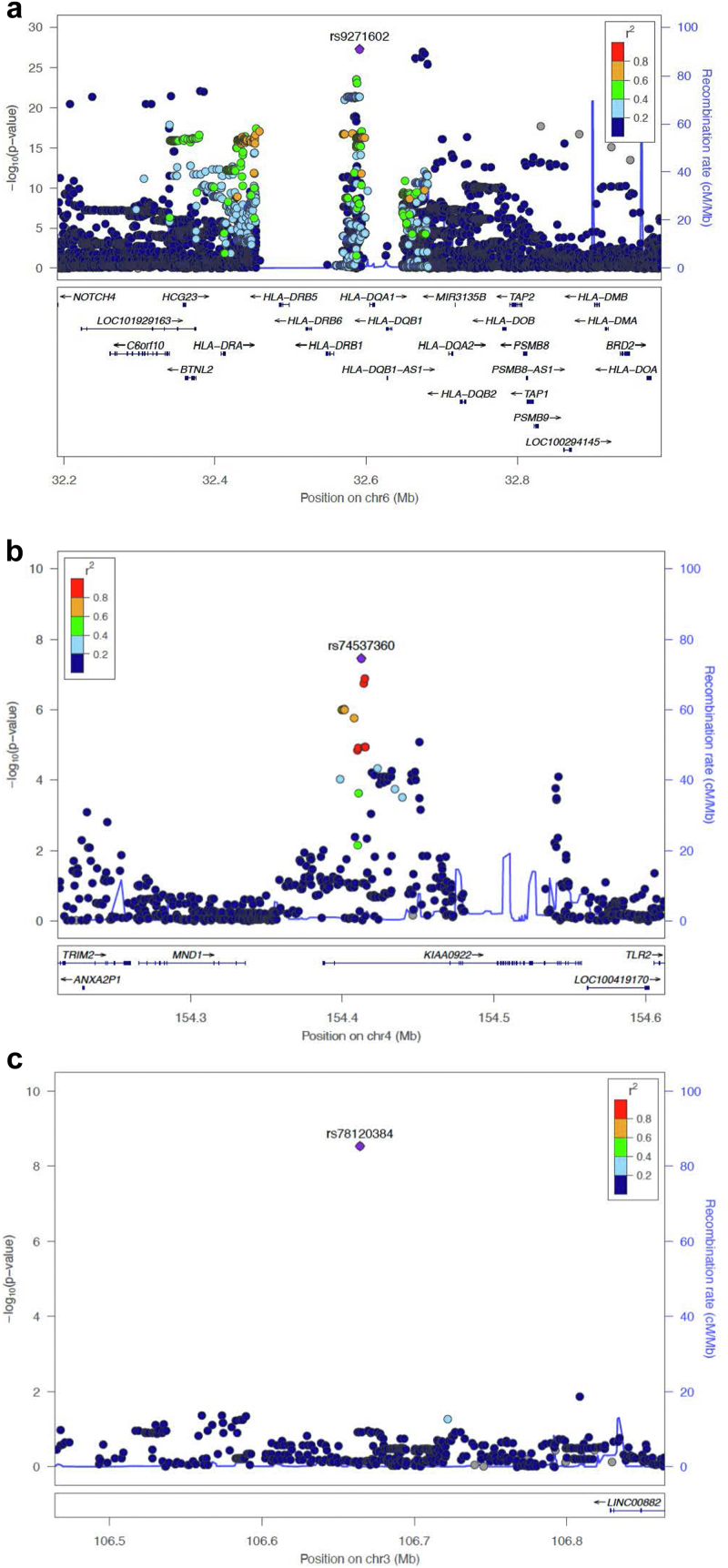
Locus zoom plot for regions on chromosomes 6p21.32 and 4q31.3 identified in the Sri Lankan discovery cohort. Index SNPs are annotated as a purple diamond over the respective genes, listed below. The surrounding SNPs colored in yellow and green are in LD with the index SNP as depicted by the r^2^ value in the legend. Genes and positions in megabases (Mb) are listed along the x-axis. The level of significance is depicted along the y-axis as −log_10_(P). Each dot represents a variant. (a) Lead SNP (rs9271602) in HLA-DQ/DR region; (b) lead SNP (rs74537360) in the KIAA0922 (otherwise known as TMEM131L) gene; and (c) lead SNP (rs78120384), upstream of the gene LINC00882. No other SNPs in LD with rs78120384 were associated with disease, suggesting a false-positive result. LD, linkage disequilibrium; SNP, single nucleotide polymorphism.

GWAS of the Sri Lankan population showed 2 independent genome-wide significant signals (Figure 2 and Table 1). The strongest association was detected in *HLA-DQA1* (rs9271602, *P* = 1.12 × 10^−27^, OR = 2.75, 95% confidence interval [CI] 2.29–3.30) (Figure 3a). Conditional analysis on rs9271602 revealed a second independent signal at rs9391784 (*P* = 1.16 × 10^−15^); further conditioning on rs9271602 and rs9391784 revealed a third signal at rs17212846 (*P* = 2.57 × 10^−13^); and conditioning on rs9271602, rs9391784, and rs17212846 revealed a fourth signal at rs9260172 (*P* = 3.27 × 10^−9^) (Supplementary Figure S3).

**Table 1:** Lead SNPs associated with Sri Lankan SSNS

SNP	Locus	Gene	DR^2^	Test Allele	AF Cases	AF Controls	OR	95% CI	*P*-Value
rs9271602	6p21.32	*HLA-DQA1*	1	G	0.53	0.29	2.75	2.29-3.30	1.12 × 10^−27^
rs78120384	3q13.12	Intergenic	1	A	0.18	0.09	2.34	1.76-3.09	2.91 × 10^−9^
rs74537360	4q31.3	*TMEM131L*	1	T	0.14	0.07	2.37	1.75-3.22	2.98 × 10^−8^

AF, allele frequency; CI, confidence interval; DR^2^, imputation dosage R^2^; OR, odds ratio; SNP, single nucleotide polymorphism.

The lead SNP at *HLA-DQA1* is in strong LD with rs2858317 and rs3828799 (identified by Dufek *et al.*^3^), rs4642516 (identified by Jia *et al.*^4^), rs1129740 and rs1071630 (identified by Gbadegesin *et al.*^11^), and rs1063348 and rs28366266 (identified by Debiec *et al.*^5^).

The next strongest association was outside the HLA region at 4q31.3 in the gene, *TMEM131L*, previously called *KIAA0922* (rs74537360, *P* = 2.98 × 10^−8^, OR = 2.37, 95% CI 1.75–3.22) (Figure 3b). Genome-wide significance was lost after conditioning on rs74537360 (Supplementary Figure S4). A further isolated marker (rs78120384) outside of HLA reached genome-wide significance, which was on the lower border of accepted allele frequencies and was deemed a false-positive result (Figure 3c).

The power of this GWAS exceeded 80% to detect common alleles (minor allele frequency >0.01) with genotypic relative risk >2.2 at a significance threshold of *P* > 5 × 10^−8^ under an additive model. The inflation factor (λ) was calculated to be 1.00 suggesting no evidence of genomic inflation.

### HLA Fine-Mapping

Significant association with SSNS was detected in 6 classical *HLA* alleles, including 3 previously reported subtypes associated with SSNS in Europeans: *DQB1∗02:01*, *DQA1∗01*, and *DQA1∗02:01*^3^ (Table 2^3^^,^^26^^,^^27^). The strongest association was observed in *DQB1∗02:01*, which was a risk haplotype. The strongest protective allele was in *HLA-DQA1∗01*. Conditional analysis on the lead HLA allele, *HLA-DQB1∗02:01,* revealed that the only further independent signal was in *HLA-B∗52:01* (Table 2^3^^,^^26^^,^^27^ and Supplementary Figure S5).

**Table 2:** Classical HLA alleles and HLA amino acid polymorphisms associated with Sri Lankan SSNS

Gene	Variants^a^	OR	95% CI	AF Cases	AF Controls	Conditional *P* value^b^	*P* value	AF EUR	AF JAP
HLA_DQB1	*HLA_DQB1∗02:01*	2.24	1.77–2.84	0.36	0.17	2.59 × 10^−11^	2.59 × 10^−11^	0.21^c^	0.01
	*HLA_DQB1 A74*	2.26	1.79–2.87	0.36	0.16	1.44 × 10^−11^	1.44 × 10^−11^	-	-
	*HLA_DQB1∗05*	0.57	0.44–0.74	0.15	0.25	1.22 × 10^−2^	3.90 × 10^−5^	0.15^d^	0.07
	*HLA_DQB1 L-4*	0.54	0.42–0.71	0.20	0.37	4.10 × 10^−6^	8.69 × 10^−11^	-	-
HLA_DQA1	*HLA_DQA1∗01*	0.59	0.48–0.72	0.33	0.52	2.60 × 10^−2^	5.92 × 10^−7^	0.36^c^	0.07
	*HLA_DQA1 L69*	1.89	1.54–2.33	0.63	0.42	1.84 × 10^−3^	1.47 × 10^−9^	-	-
	*HLA_DQA1∗02:01*	1.72	1.37–2.17	0.36	0.18	6.35 × 10^−1^	3.99 × 10^−6^	0.15^c^	0.04
HLA_DRB1	*HLA_DRB1 R4*	1.78	1.42–2.23	0.39	0.20	1.67 × 10^−1^	5.64 × 10^−7^	-	-
HLA_DPB1	*HLA_DPB1∗17:01*	4.04	2.10–7.77	0.03	0.01	3.56 × 10^−3^	2.80 × 10^−5^	0.009^e^	0.01
HLA_A	*HLA-A DHR114*	1.64	1.32–2.03	0.43	0.25	4.00 × 10^−3^	5.81 × 10^−6^	-	-
HLA_B	*HLA_B∗52:01*	2.00	1.37–2.91	0.08	0.08	2.00 × 10^−5^	2.98 × 10^−4^	0.03^d^	0.11

AF, allele frequency; AF, allele frequency; CI, confidence interval; EUR, European; JAP, Japanese; OR, odds ratio.

a We identified independent associations for each category of variants: HLA classical allele and HLA amino acid. For amino acid polymorphism, the label specifies the amino acid and position. For example, “HLA_DQB1 A74” means amino acid Alanine at position 74 of the HLA_DQB1 protein.

b The “Conditional *P* value” column contains conditional p values generated after iterative conditional regression within each category of variant (HLA alleles and HLA amino acid polymorphisms). The “*P* value” column contains the unconditional association test *P* values.

c Values obtained from Dufek *et al*.^3^

d Values obtained from Tokić *et al*.^27^

e Values obtained from Lemin *et al*.^26^ All allele frequency for the Japanese population were obtained from http://hla.or.jp.

Four *HLA* amino acid polymorphisms were significantly associated with disease, with the strongest association observed with an Alanine residue at position 74 of the HLA-DQB1 protein, which was a risk allele. Conditional analysis revealed a further protective allele with a Leucine substitution at position −4 of the *HLA-DQB1* protein (Table 2^3^^,^^26^^,^^27^)*.*

### Transethnic Meta-Analysis

**Figure 4:**
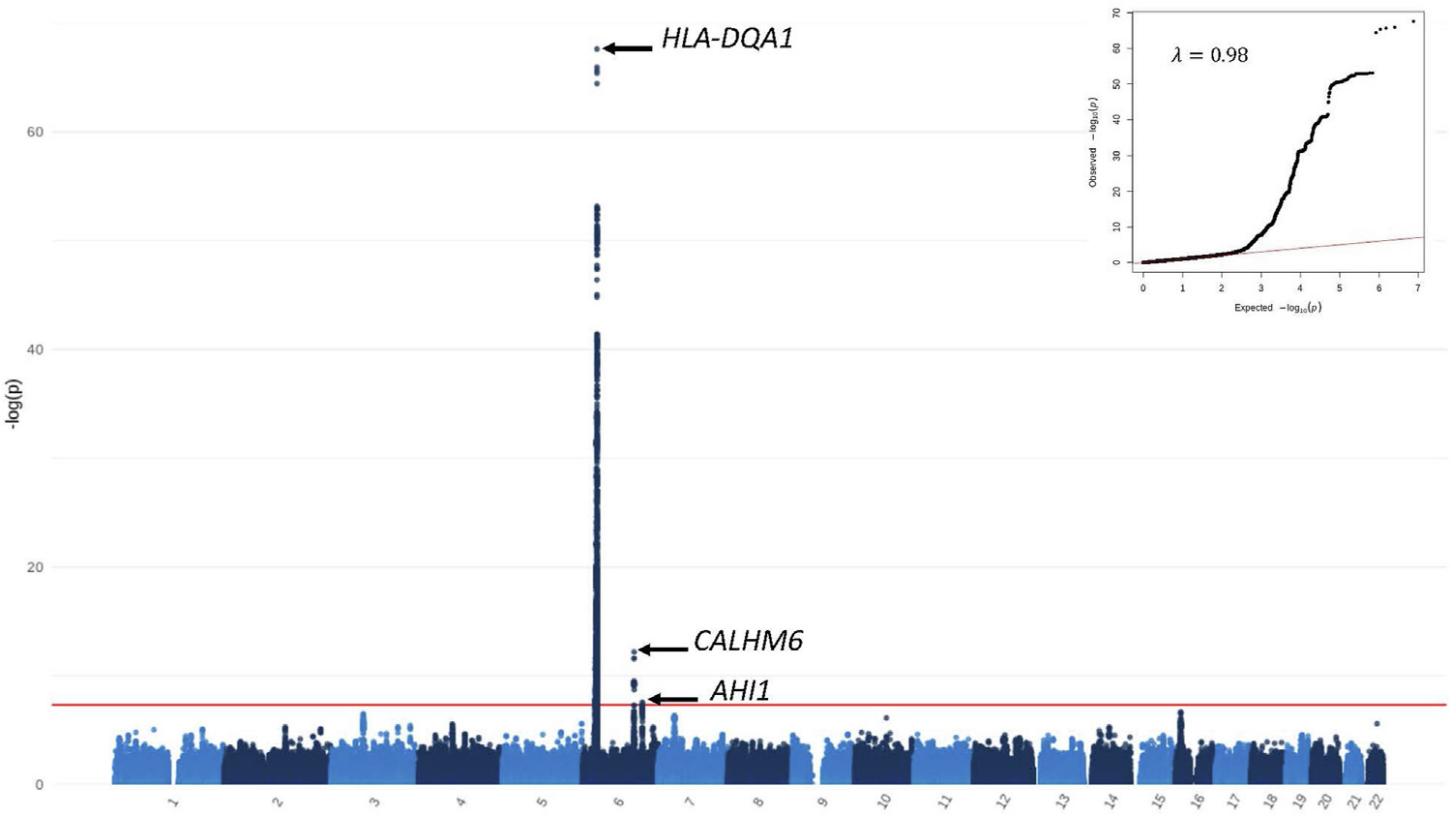
Manhattan plot of the transethnic meta-analysis of the Sri Lankan discovery cohort and the European replication cohort. Autosomal chromosomes (1–22) are listed along the x-axis. The level of significance is depicted along the y-axis as −log_10_(P). Each dot represents a variant. The red line represents the threshold of genome-wide significance (P = 5 × 10^−8^). The inverse-variance method based on a fixed-effects model was used. Three loci achieve genome-wide significance on chromosome 6; variants in HLA-DQA1, CALHM6, and AHI1 are labeled on the plot. QQ-plot and lambda are displayed in the top right corner.

**Figure 5:**
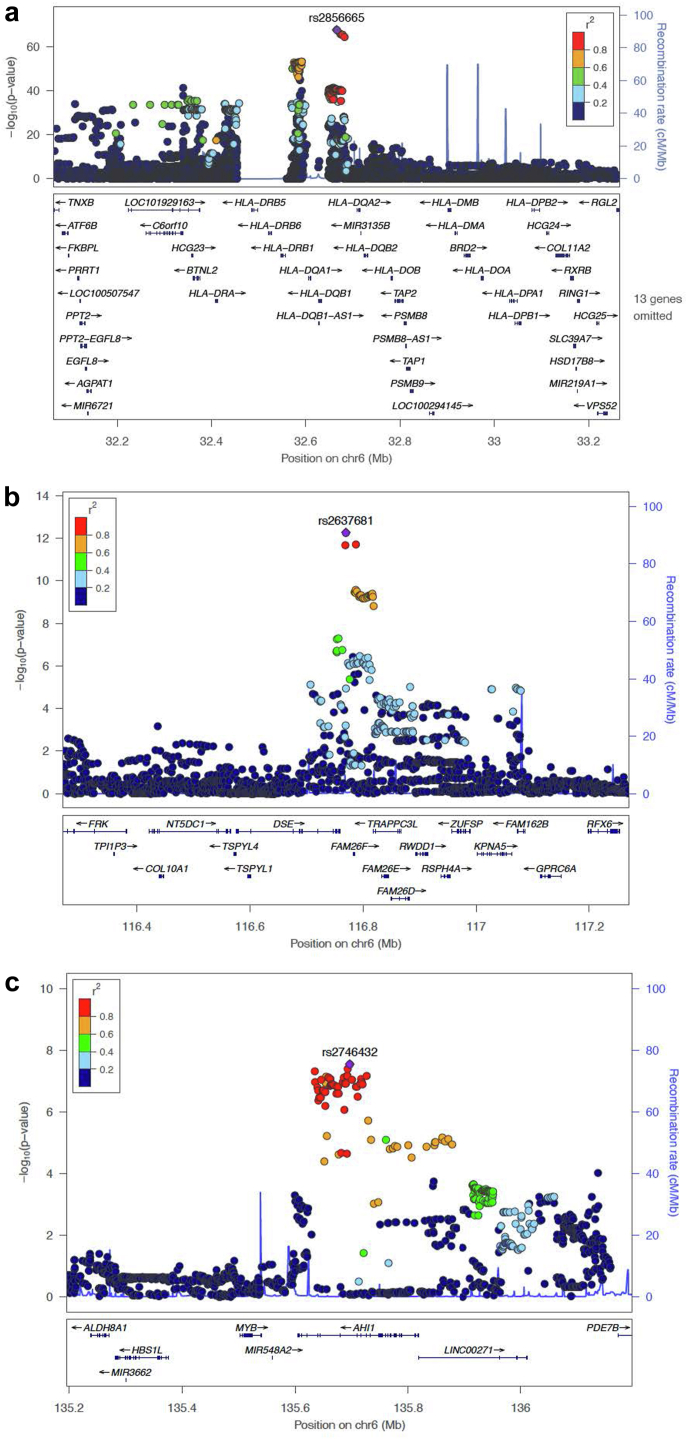
Locus zoom plot for regions on chromosomes 6p21.32, 6q22.1, and 6q23.3 in transethnic meta-analysis. Index SNPs are annotated as a purple diamond over the respective genes, listed below. The surrounding SNPs colored in yellow and green are in linkage disequilibrium with the index SNP as depicted by the r^2^ value in the legend. Genes and positions in megabases (Mb) are listed along the x-axis. The level of significance is depicted along the y-axis as −log_10_(P). Each dot represents a variant. (a) Lead SNP (rs2856665) in HLA-DQ/DR region; (b) lead SNP (rs2637681) in the FAM26F (otherwise known as CALHM6) gene; and (c) lead SNP (rs2746432) in the AHI1 gene. SNP, Single nucleotide polymorphism.

Transethnic meta-analysis was performed with the previously reported European GWAS by Dufek *et al.*,^3^ and showed strongest association at the *HLA-DQ/DR* locus (rs2856665, *P* = 2.45 × 10^−68^, OR = 4.06, 95% CI 3.47–4.75). The European-identified signal in 6q22.1 (*CALHM6*) was also detected, though it was primarily driven by the European cohort (rs2637681, *P* = 6.69 × 10^−13^, OR = 0.62, 95% CI 0.54–0.71). A novel association, driven by a combination of both cohorts, was identified at 6q23.3 in the gene *AHI1* (rs2746432, *P* = 2.79 × 10^−8^, OR = 1.37, 95% CI 1.22–1.52). See Table 3, Supplementary Table S1, and Figures 4 and 5a–c. The signal in *TMEM131L* was not replicated, though the set of overlapping markers used in the meta-analysis did not include the lead SNP at this locus.

**Table 3:** Lead SNPs associated with SSNS in Sri Lankan-European meta-analysis of childhood SSNS

SNP	Locus	Gene	Test Allele	I^2^	*P*_heterogeneity	OR	95% CI	*P*-value
rs2856665	6p21.32	*HLA-DQB1/DQA2*	G	0.00	5.58 × 10^−1^	4.06	3.47–4.75	2.45 × 10^−68^
rs2637681	6q22.1	*CALHM6*	G	92.5	2.67 × 10^−4^	0.62	0.54–0.71	6.69 × 10^−13^
rs2746432	6q23.3	*AHI1*	C	0.00	8.19 × 10^−1^	1.37	1.22–1.52	2.79 × 10^−8^

CI, confidence interval; I^2^, percentage of total variation across studies because of heterogeneity; P_heterogeneity, P-value of Cochrane Q test for heterogeneity; SNP, single nucleotide polymorphism.

### Replication

The novel genome-wide significant signal at 6q23.3 *(AHI1)* was replicated in an independent South Asian population (INSIGHT cohort) (rs2746432, *P* = 1.13 × 10^−2^, OR = 1.58), although the power to do so was only 0.466 (Table 4). This lead SNP also showed evidence of association in the Japanese cohort published by Jia *et al.* (rs2746432, *P* = 1.08 × 10^−3^).^6^ The signal at 4q31.3 (*TMEM131L)* was not replicated in this cohort (rs74537360, *P* = 0.76, OR = 1.09), despite power to detect this signal being 0.894.

**Table 4:** Replication of candidate SNPs associated with Sri Lankan SSNS in INSIGHT cohort

SNP	Locus	Gene	Test Allele	AF Cases	AF Controls	OR	95% CI	*P*-value
rs2746432	6q23.3	*AHI1*	C	0.52	0.41	1.56	1.10–2.26	1.13 × 10^−2^
rs74537360	4q31.3	*TMEM131L*	T	0.09	0.09	1.09	0.62–1.93	0.76

AF, allele frequency; CI, confidence interval; OR, odds ratio; SNP, single nucleotide polymorphism.

### Gene Annotation

The lead SNP at the 6q23.3 locus (rs2746432) is a protein-coding variant for *AHI1* and exhibits cis-eQTLs in the GTEx^28^ database in almost all of the 54 tissues tested. Notably, rs2746432 shows strong cis-eQTL effects in fibroblasts (normalized effect size (NES) 0.51, *P* = 1.6 × 10^−28^), Epstein-Barr virus-transformed lymphocytes (NES 0.60, *P* = 2.6 × 10^−9^), and in the spleen (NES 0.41, *P* = 6.0 × 10^−9^). The SSNS risk (minor) allele in rs2746432 decreased the expression of *AHI1* in all cell types, indicating that in cases where the risk allele was more frequent, the expression of *AHI1* is down-regulated. The lead SNP (rs2746432) is also an eQTL for the genes *LINC00271* (nonprotein-coding variant) with strongest effects in testis (NES 0.15, *P* = 4.1 × 10^−6^) and thyroid (NES 0.14, *P* = 9.0 × 10^−8^) and *RP3-388E23.2* (novel transcript in noncoding gene) with strongest effects in the cerebellum (NES 0.29, *P* = 7.7 × 10^−6^) and pituitary gland (NES 0.26, *P* = 2.7 × 10^−5^). Gene annotation in the UCSC genome browser demonstrates that the promoter region of *AHI1* and *LINC00271* overlap, and that when *AHI1* is turned on, *LINC00271* is turned off.^29^

In the Human Kidney Cell Atlas, *AHI1, LINC00271,* and *RP3-388E23.2* did not show any significant expression in adult kidney-related tissues; however, in the fetal kidney, *AHI1* expression was significant in many kidney tissues, and was highest in the proximal tubule and plasmacytoid dendritic cells (a cell type that specializes in interferon production).^30^ There was no significant eQTL for rs2746432 in the Human Kidney eQTL Atlas.^31^

## Discussion

The present Sri Lankan GWAS and transethnic meta-analyses were performed in the largest South Asian cohort to date and identified common variants in *AHI1* as a new susceptibility locus for childhood SSNS. This study has also confirmed previous association findings of SSNS with *HLA-DQ/DR.* Furthermore, the larger sample size enabled additional fine-mapping of the *HLA* locus. These findings provide new insights into our understanding of the genetic background of childhood SSNS, and further support an immunologic basis to its pathogenesis. The identification of *AHI1* and its associated proteins also reveals new targets for biological inquiry and potential therapeutic development, and provides evidence that genes implicated in rare Mendelian disorders can also harbor common variants in a complex disease.

Gene annotation of the lead SNP at the 6q23.3 locus (*AHI1)* revealed that this SNP has eQTL effects in *AHI1*, *LINC00271*, and *RP3-388E23.2*. Indeed, in the meta-analysis, the lead SNP demonstrated LD extending into *LINC00271*, and functional annotation observed the transcription start site of these 2 genes to be overlapping, suggesting the potential for coregulation of these genes. Previous studies have indeed reported association of disease traits with an LD block encompassing all 3 genes.^32^^,^^33^

*AHI1* encodes the protein, jouberin, which is a component of a ring-like protein complex in the transition zone at the base of cilia.^34^ Together, with the other proteins that compose the complex, *AHI1* acts to restrict protein diffusion between the plasma and ciliary membranes; disruption of the complex leads to reduction in cilia formation and a reduction in signaling receptors from the remaining cilia.^35^ Rare biallelic mutations in *AHI1* cause Joubert syndrome, a rare monogenic disorder manifesting in agenesis of the cerebellum, ataxia, hypotonia, and intellectual disabilities.^36^ Interestingly, our meta-analysis revealed that common variants in *AHI1* are associated with SSNS.

*AHI1* has a diverse array of biological functions. It is known to be important in the kidney through its interaction with *NPHP1*, which encodes another protein at the basal body of cilia. Mutations in *NPHP1* are associated with Joubert syndrome accompanied by renal dysfunction, accounting for the majority of cases of nephronophthisis.^37^ *AHI1* and *NPHP1* form heterodimers and heterotetramers, and mutations in *AHI1* have been shown to change this binding pattern.^36^

*AHI1* is also involved in immune system function. Jiang *et al.* found that *AHI1* is highly expressed in primitive types of normal hematopoietic cells and is down-regulated during early differentiation.^38^ Therefore, alterations in *AHI1* expression may contribute to the development of certain types of human leukemias. Notably, a GWAS in the autoimmune disease multiple sclerosis detected a susceptibility variant in *AHI1* (rs4896153) (with LD extending into *LINC00271*) that was subsequently shown to have strong *cis-*eQTL effect on overall *AHI1* expression.^32^ Functional studies showed that expression peaked after stimulation of human CD4+ T cells, suggesting that it may play a role in early T-cell receptor activation. *AHI1* has also been shown to be involved in actin organization,^39^ and therefore the authors of this study speculated that *AHI1* may play a role in the formation or stabilization of the T-cell receptor synapse as a mechanism for its association with multiple sclerosis.^32^

The eQTL analysis of the lead SNP, rs2746432, showed *cis*-eQTL effects on *AHI1* in Epstein-Barr virus-transformed lymphocytes, with the risk allele at this variant associated with decreased *AHI1* expression. Thus, it is possible that decreased *AHI1* expression in the lymphocytes of individuals with SSNS could lead to increased cytokine production, and/or destabilization of the T-cell receptor complex, both resulting in immune system dysregulation. The association of common variation at *AHI1* with SSNS in addition to the established association of (biallelic) rare variants of *AHI1* with Joubert syndrome demonstrates that variation across the allele frequency spectrum in a gene can contribute to both monogenic and polygenic disease, and that these alleles might act by different mechanisms, resulting in altogether different disorders.

The strongest association in our analysis was in the HLA region. In the Sri Lankan discovery cohort, the lead SNP, rs9271602, was in the *HLA-DR/DQ* region, specifically in the *HLA-DQA1* and *HLA-DQB1* genes. This finding was also detected in the previously published European GWAS,^3^ and was therefore unsurprisingly also observed in our transethnic meta-analysis of the European and Sri Lankan cohorts, with the strongest association at rs2856665, between *HLA-DQB1* and *HLA-DQA2* but with LD extending to *HLA-DQA1*. All previous GWAS published on SSNS have found association within these genes, including populations of European, South Asian, and Japanese ancestry.^3^, ^4^, ^5^, ^6^^,^^40^

Fine-mapping of the HLA alleles identified *HLA-DQB1∗02:01, HLA-DQA1∗02:01, HLA-DPB1∗17:01*, and *HLA-B∗52:01* to be associated with increased risk of SSNS. Of these, *HLA-DQA1∗02:01* and *HLA-DQB1∗02* were also found to be associated with increased risk of disease in European and South Asian studies.^3^^,^^5^^,^^40^ *HLA-DQA1∗01* and *HLA-DQB1∗05* were the protective alleles associated with SSNS in our Sri Lankan discovery cohort, with the *HLA-DQA1∗01* allele replicating in European^3^ and South Asian^40^ populations. Conversely, in Japanese populations, altogether different risk and protective HLA alleles have been identified.^4^^,^^6^ These findings demonstrate substantial overlap between European and South Asian populations, but not Japanese. This is most likely explained by differing allele frequencies in the different populations (see Table 2 and Supplementary Table S2).

In the conditional analysis on the lead allele, *HLA-DQB1∗02:01*, in our Sri Lankan discovery cohort, both the risk and protective alleles at *HLA-DR/DQ* disappeared, leaving only *HLA-B∗52:01* as the independent signal. *HLA-B∗52:01* (the most common subtype of allele at B∗52) was the only class I HLA allele associated with disease in our discovery GWAS analysis. Although this allele was relatively rare in the Sri Lankan population (minor allele frequency 0.08), it is interesting because of its association with several other immune-mediated diseases, including ulcerative colitis^41^ and Takayasu’s arteritis.^42^

Association analysis of the amino acid polymorphisms revealed the strongest association to be a risk allele at position 74 in the *HLA-DQB1* protein. *HLA-DQB1 A74* is involved in the formation of the peptide-binding cleft,^43^ and it is also in high LD with *HLA-DQB1 A57* which is critical for peptide binding and recognition.^44^

The strongest association outside of HLA in our transethnic meta-analysis was on chromosome 6 (rs2637681, *P* = 5.44 × 10^−13^, OR = 0.62, 95% CI 0.54–0.70) in the gene, *CALHM6*.^45^ This locus was associated with SSNS in the previously published European GWAS,^3^ and was also reported as a potential signal in the SSNS GWAS by Debiec *et al.*^5^ However, it was not significantly associated with disease in our Sri Lankan discovery cohort (Supplementary Table S1). Unsurprisingly, association of rs2637681 in our transethnic meta-analysis was mainly driven by the European cohort. The direction of effect was the same in both cohorts, however, which supports the relevance of this finding.

Power calculation indicated that the Sri Lankan discovery GWAS was powered to detect a signal of similar strength at this locus at *P* < 0.05, even considering the lower frequency of the associated allele in the Sri Lankan population (0.162 compared with 0.413 in Europeans). However, the observed signal (*P* = 0.199, OR = 0.86, 95% CI 0.69–1.08) was not as strong as this. There are several potential explanations for this, including differences in LD patterns in individuals of different ancestries, or that there is a true difference in effect size at this variant, perhaps because of differences in genetic background or environmental exposures.^46^ It has been previously demonstrated that variants associated with a particular disease in one ancestral group are not always reproduced in another.^47^ Furthermore, variants shared among autoimmune disorders have been shown to be protective in one disorder and risky in another.^48^

This study has several limitations. First, there was limited clinical information on the individuals included in the study. Details such as age of onset or relapse pattern could have enhanced our understanding of the relationship between markers of clinical severity and number of risk alleles, although the relatively small size of the cohort would limit the power to perform this type of analysis. Second, the case and control data sets were genotyped on different platforms, which limited the number of overlapping markers. We overcame this problem by filtering for genotyping discrepancies and imputing the data sets separately, but this has greater potential for error than if the case and control data sets were genotyped on the same platform. Third, though this study provides evidence of association with alleles at *TMEM131L* in the Sri Lankan discovery cohort*,* this was not replicated in either the meta-analysis or in an independent South Asian cohort, suggesting that this association is most likely a type 1 error; it is also possible that this association represents a genetic risk factor uniquely found in the Sri Lankan (as opposed to the South Asian or European) population. Replication in an independent Sri Lankan cohort is needed.

In summary, our study showed a novel association of childhood SSNS with alleles at *AHI1* and confirmed previous associations at *HLA-DR/DQ.* These findings further support the role of immune dysregulation in the pathophysiology of disease. The *AHI1* association, in particular, suggests a link between a ciliary gene and glomerular disease and reinforces an emerging paradigm in nephrology: in genes harboring rare Mendelian variants, common alleles can increase the susceptibility of polygenic diseases. Our study also illustrates the importance of performing GWAS in larger data sets by combining populations of diverse ancestry, because by doing so, we were able to increase the power to detect a novel variant associated with SSNS.

## Disclosure

All the authors declared no competing interests.
