# Supplementary material for: Common Risk Variants in AHI1 Are Associated With Childhood Steroid Sensitive Nephrotic Syndrome
Source: Kidney Int Rep. 2023 May 27;8(8):1562–74. doi: 10.1016/j.ekir.2023.05.018 (PMC10403666; doi:10.1016/j.ekir.2023.05.018)
Supplement: Supplementary Methods [file mmc1.pdf]

SUPPLEMENTARY MATERIAL

Common risk variants in *AHI1* are associated with childhood steroid-sensitive nephrotic syndrome

Mallory L Downie[1,2], Sanjana Gupta[1], Catalin Voinescu[1], Adam P Levine[3], Omid Sadeghi-Alavijeh[1], Stephanie Dufek-Kamperis[4], Jingjing Cao[5], Martin Christian[6], Jameela A Kari[7], Shenal Thalgahagoda[8], Randula Ranawaka[8], Asiri Abeyagunawardena[8], Rasheed Gbadegesin[9], Rulan Parekh[5,10], Robert Kleta*[1,2], Detlef Bockenhauer*[1,2], Horia C Stanescu*[1], Daniel P Gale*[1]

[1]Department of Renal Medicine, University College London, London, UK
[2]Paediatric Nephrology, Great Ormond Street Hospital for Children NHS Foundation Trust, London, UK
[3]Department of Pathology, University College London, London, UK
[4]Department of Paediatrics and Adolescent Medicine, Aarhus University Hospital, Aarhus, Denmark
[5]Division of Nephrology, Department of Pediatrics, The Hospital for Sick Children, Toronto, Canada
[6]Nottingham Children's Hospital, Nottingham, UK
[7]Pediatric Nephrology Centre of Excellence, King Abdulaziz University, Jeddah, Kingdom of Saudi Arabia
[8]Department of Paediatrics, University of Peradeniya, Peradeniya, Sri Lanka
[9]Department of Pediatrics, Duke University School of Medicine, Durham, North Carolina, USA
[10]Department of Medicine, Women's College Hospital, Toronto, Canada

Address for correspondence/Request for reprints:

Professor Daniel P Gale

UCL Department of Renal Medicine

1st Floor, Royal Free Hospital

Rowland Hill Street

London NW3 2PF

United Kingdom

Email d.gale@ucl.ac.uk

Table of Contents

Supplementary Methods 3
Case definition 3
Case cohort description 3
Genotyping case cohort 3
Post-genotyping processing 4
Quality control 4
Whole-Genome Imputation 5
GWAS Methods and conditional analysis 6
HLA Imputation 7
HLA Fine-mapping 7
Trans-ethnic meta-analysis 8
Replication 8
Functional gene annotation 8
Supplementary Figures 9
Supplementary Tables 15
Supplementary References 17

Supplementary Methods

Case definition

All cases included in the study had childhood onset idiopathic nephrotic syndrome as defined by KDIGO(1): proteinuria (40 mg/m^2/day or urine protein/creatinine ratio > 200mg/mL or 3+ protein on urine dipstick), hypoalbuminemia (<25 g/L), and edema. Steroid sensitive nephrotic syndrome was defined as response to corticosteroid steroid treatment within four weeks(1). Patients with steroid resistant (defined as no response to corticosteroids within four weeks of therapy) or with secondary causes of nephrotic syndrome were excluded from the study.

Case cohort description

A total of 663 individuals of self-reported South Asian ancestry with SSNS were collected by the authors, including those from the PREDNOS (EudraCT 2010-022489-29) and PREDNOS2 (EudraCT 2012-003476-39) trials(2,3). After selection for genetic Sri Lankan ancestry, 420 cases remained; 162 were female and 250 were male, in keeping with the reported 1:2 female:male ratio of childhood SSNS(4).

Genomic DNA was extracted from peripheral blood following a standard protocol. Consent for DNA collection and analysis was obtained from each participant by each collaborator and ethical approval was granted by host institutions according to local policies.

Genotyping case cohort

Genotyping of all individuals with SSNS was performed at UCL Genomics (Institute of Child Health, UCL, London, UK) using the Illumina Infinium Multi-Ethnic Global BeadChip v.A1 with 1,779,818 markers. Sample processing was performed in accordance with the Infinium HD

Ultra Assay protocol (Part # 15023140 Rev. A, Illumina Inc, San Diego, USA). First, 200ng DNA was whole genome amplified (37°C for 1 hour and 15 minutes), precipitated and resuspended in hybridization buffer. Samples were hybridized onto Illumina array BeadChips using a liquid handling robot (Freedom Evo, Tecan Ltd, Switzerland), and then scanned using the iScan with autoloader (Illunima, San Diego, USA). Data was then collected in raw IDAT format(5).

Post-genotyping processing

An in-house program was utilized to re-encode genotypes from the Illumina encoding scheme to the forward/reverse encoding scheme using the Illumina manifest file and dbSNP version 151 as a reference(6). This software excluded indels, CNVs, and genetic variants on the sex chromosomes. After this processing, 730,728 autosomal variants remained.

Quality control

Prior to imputation, quality control was performed on the case and control cohorts separately following a standard protocol (see Figure 1)(7). In the case dataset, individuals were removed for low call rate of <95% (n=46), low genotyping quality (heterozygosity rate +/- 3 standard deviations from the mean (n=9)), and relatedness (IBD≤0.1875 using KING-robust kinship estimator(8), n=147). Single nucleotide polymorphisms (SNPs) were excluded based on being multiallelic, low call rate <99% (80,300 markers) and low minor allele frequency (MAF) <0.01 (200,804 markers). In the UK Biobank control dataset, individuals were excluded for call rate <95% (n=6), heterozygosity rate +/- 3 standard deviations from the mean (n=24), and relatedness (IBD<0.1875, n=459). Markers were excluded by more than two alleles, low call rate <99% (112,159 markers), low MAF<0.01 (190,273 markers), and deviation from the Hardy-

Weinberg equilibrium ($p<0.01$, 298,522 markers). A further filter was applied to both cohorts to remove SNPs genotyped discrepantly between the MEGA BeadChip and Axiom arrays. These SNPs were identified by comparison (basic allele test) of a separate group of Sri Lankan healthy control subjects (n=199, obtained from collaborating clinicians) genotyped on the MEGA BeadChip and the control cohort genotyped on the Axiom array (see Supplementary Figure S1). Markers with $-\log(p)>5$ were identified by this analysis, representing the spurious associations related to plate discrepancy. These markers were then filtered from each dataset (225 markers total). Importantly, there were no overlapping individuals in the Sri Lankan healthy control cohort and the Sri Lankan SSNS case cohort of the discovery GWAS. After these filtering steps, 507 individuals and 449,427 markers remained in the case dataset and 13,299 individuals and 481,630 markers remained in the control dataset (see Figure 1).

PCA was then performed in PLINK v2.00 which generated the first ten principal components (PCs) to control for population stratification. A custom R script (https://github.com/APLevine/PCA_Matching) was then used to match cases to controls within a distance threshold calculated using these ten PCs weighted by the percentage of genetic variation explained by each component (see Supplementary Figure S2). Only controls within a user-defined specified distance from a case were included, with each case having to match a minimum of one control to be included in the final cohort. This analysis removed 87 cases and 10,960 controls, leaving 420 cases and 2339 controls in the final dataset.

Whole-Genome Imputation

Whole genome imputation was performed on the final case and control datasets, separately: there were 420 individuals and 449,427 markers in the case dataset, and 2339 individuals and

481,630 markers in the control dataset. Imputation analysis was performed using *minimac4* within the Michigan Imputation Server(9) using the 1000 Genomes Project phase 3 data as a reference panel, which included 50,008 haplotypes from 26 populations across the world, of which 386 individuals were of South Asian ancestry(10).

Imputation was performed per chromosome and the imputed data was subsequently filtered for imputation quality which removed all markers with a dosage R^2<80%. This resulted in 6,457,823 high-quality variants in the case dataset and 6,131,717 high-quality variants in the control dataset. Cases and controls were then merged to yield 7,277,381 variants, of which not all were shared between datasets. Further quality control was then performed on the combined case-control dataset to exclude further markers as follows: call rate/missingness <99% (1,965,222 markers removed), MAF <0.01 (0 markers removed), and HWE p<0.01 in controls only (47,034 markers removed). This resulted in a final dataset of 5,265,125 in 420 cases and 2339 controls. PLINK versions 1.90 and 2.00 were used for quality control analysis[19].

GWAS Methods and conditional analysis

Association analyses were conducted in SAIGE-GENE(11) using a logistic mixed model method, adjusting for sex and the first three principal components. Using more than three principal components resulted in genomic deflation (lambda <1.0), suggesting over-fitting. Conditional analysis of the lead SNPs was performed in SAIGE using the same logistic mixed model adjusted for sex and the first three principal components. A genome-wide significance threshold of $p<5x10^{-8}$ was used. R v4.2.1 was used to generate Manhattan plots. Regional plots were generated using LocusZoom with 1000 Genomes Nov 2014 used as the LD reference(12).

HLA Imputation

HLA imputation was also performed on the case and control dataset separately using the HLA-TAPAS(13) reference panel available on the Michigan Imputation Server (as described in Section 5.7), which included 489 South Asian individuals (most of which were obtained from the 1000 Genomes Project(10)). Imputation results were filtered to remove markers with dosage R^2<80% and to remove insertions/deletions. The case and control dataset were merged to yield a combined set of 46,722 high-quality variants. Quality control was then performed on the dataset to exclude markers as follows: call rate/missingness <99% (6498 variants removed), MAF <0.01 (0 variants removed), and HWE p-value <0.01 in controls (482 variants removed). This yielded a total of 39,792 variants.

HLA Fine-mapping

HLA association analysis was performed in PLINK on the dataset of 420 cases versus 2339 controls and 136 HLA 4-digit alleles initially using logistic regression adjusted for the first ten principal components of ancestry. Association testing was performed in PLINK rather than SAIGE at HLA because SAIGE's saddle point approximation requires genome-wide variance to produce an accurate value. HLA association testing was only performed on a small portion of the genome, and therefore, logistic regression analysis in PLINK was the more accurate method (see Supplementary Figure S5). Conditional analysis of the lead HLA allele was done using the same logistic regression model adjusted for sex and the first ten principal components. A significance threshold of $p<3x10^{-4}$ (0.05/136) was used to adjust for multiple comparisons with the n=136 4-digit HLA alleles used in the analysis.

Trans-ethnic meta-analysis

The trans-ethnic meta-analysis was performed with META(14), using the inverse variance method. The European dataset consisted of 422 individuals with childhood SSNS, 5642 controls, and 5,216,266 markers. The Sri Lankan dataset consisted of 420 individuals with childhood SSNS, 2339 controls, and 5,265,125 markers. There were 3,727,716 overlapping markers between these two datasets and it was only these overlapping markers that were included in the meta-analysis. Sample sizes (420 and 422) and lambda values (1.00 and 1.03) for the Sri Lankan and European datasets were included as input values, respectively.

Replication

Replication of the two novel candidate SNPs (in *TMEM131L* and *AHI1*) was tested in an independent population that comprised 150 South Asian (including Sri Lankan) participants from the INSIGHT(15) cohort and 277 controls from the Spit for Science study(16). South Asian genetic ancestry was determined by PCA using the first ten principal components calculated in PLINK v2.00 projected onto 1000 Genomes(10) ancestry controls as reference. Participants in these studies were genotyped on Infinium® Global Diversity Array-8 BeadChip and imputed using the TOPMed imputation reference panel(17). Association analyses were carried out under an additive model. Significance threshold for replication was considered as $p<0.05/2=2.50x10^{-2}$.

Functional gene annotation

Publicly available databases such a GTEx(18), the Human Kidney Cell Atlas(19), the Human Kidney eQTL Atlas(20) and the UCSC Genome Browser(21) containing gene expression data and expression quantitative trait loci (eQTLs) were used to determine whether tissue-specific

gene expression was altered by any of the variants found to be associated with SSNS and in what tissues the genes closest to the associated variants were expressed.

Supplementary Figures

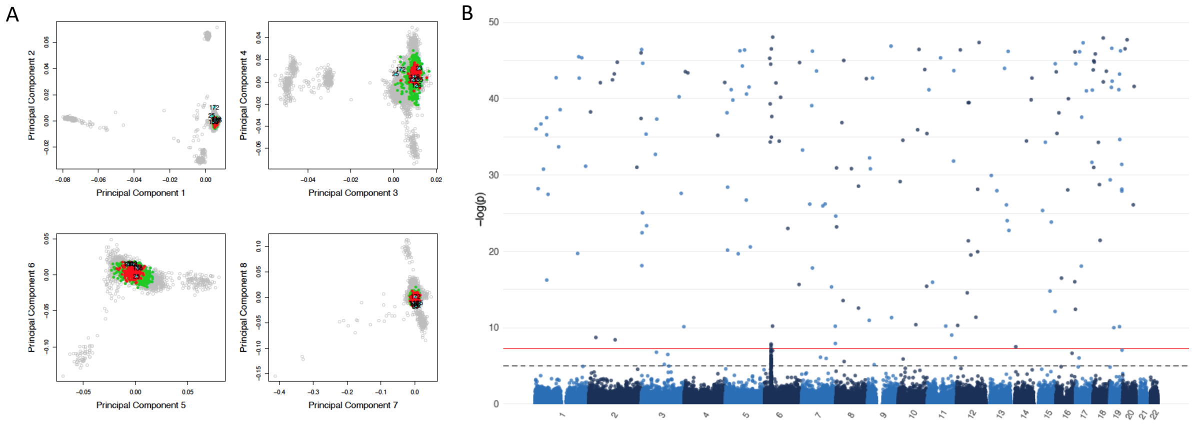

Supplementary Figure S1: Genotyping discrepancy analysis in Sri Lankan controls genotyped alongside Sri Lankan SSNS cases versus UK Biobank controls

A) Principal component analysis of an independent Sri Lankan control cohort (n=191, in red) and ancestry-matched UK Biobank controls (n=672 in green). Each of the four panels in this figure displays two principal components compared to one another (principal component 1 versus 2 in the top left panel, and so on). Plots were generated using the custom R script discussed in the Supplementary Methods. Each dot represents an individual. Sri Lankan controls are depicted in red, ancestrally matched UK Biobank controls are depicted in green, and unmatched individuals are depicted in grey (UK Biobank) or blue with black numbers (Sr Lankan). Unmatched individuals (grey or blue) were removed from the dataset.

B) Manhattan plot of logistic regression analysis of Sri Lankan Controls versus UK Biobank controls. Autosomal chromosomes (1-22) are listed along the x axis. The level of significance is depicted along the y axis as -$\log_{10}$(p). Each dot represents a variant. The red line represents the threshold of genome-wide significance (p=5×10^{-8}). The dashed line represents the threshold for suggestive genome-wide association (p=1×10^{-5}). All variants above the suggestive genome-wide significance threshold (n=225) were removed from the discovery cohort case-control dataset.

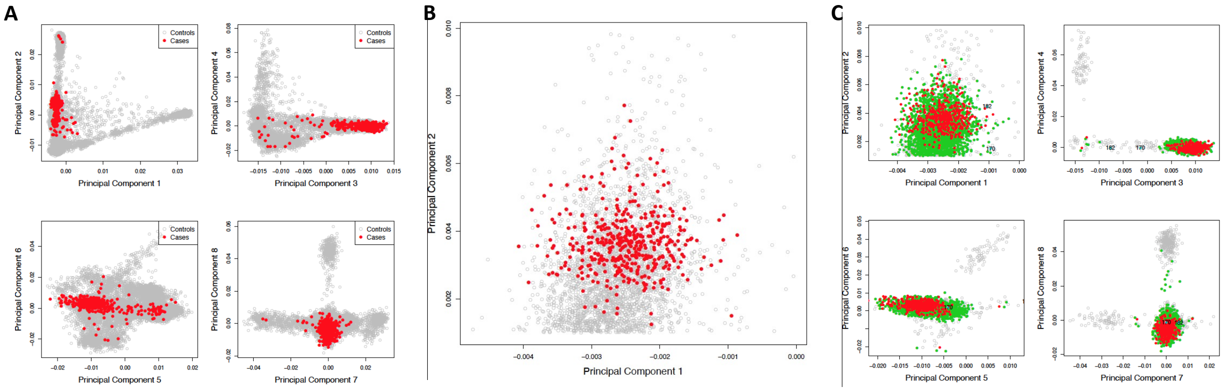

Supplementary Figure S2: Principal component analysis of case-control dataset anchored by 1000 Genomes controls

Each panel in this figure displays two principal components compared to one another (principal component 1 versus 2 in the top left panel, and so on). Plots were generated using the custom R script discussed in the Supplementary Methods. Each dot represents an individual. Cases are depicted in red, controls are depicted in grey, and matched controls are depicted in green. Controls are a combination of 1000 Genomes controls and UK Biobank controls. Many controls do not overlap with the cases and were therefore eliminated from the analysis.

A) Initial principal component analysis showing data plotted for principal components 1-8 in 4 respective graphs; B) An initial filter was applied to case-control dataset to include cases of Sri Lankan ancestry only (in red); C) Principal component analysis showing selected controls (in green) matched to cases (in red). Blue dots with black labels indicate cases with an insufficient number or closeness of an appropriate control (optimally user-defined) and therefore these cases were eliminated from the dataset.

A. Logistic Regression

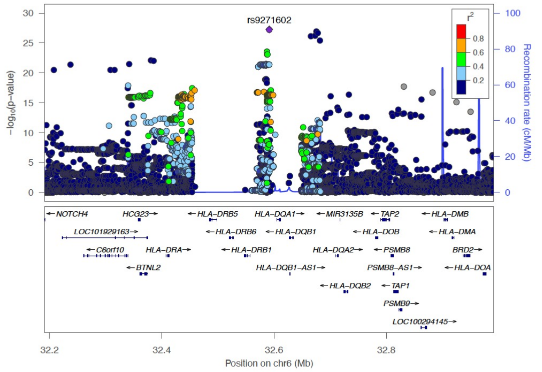

B. Conditional analysis on rs9271602

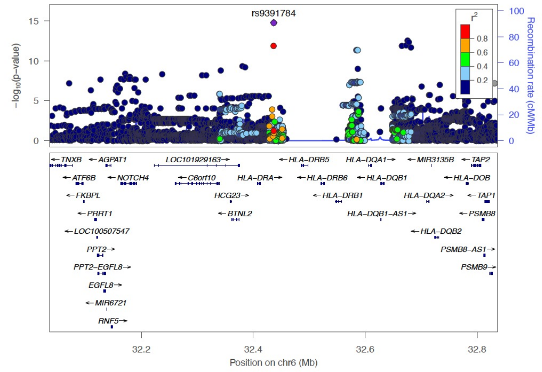

C. Conditional analysis on rs9271602 & rs9391784

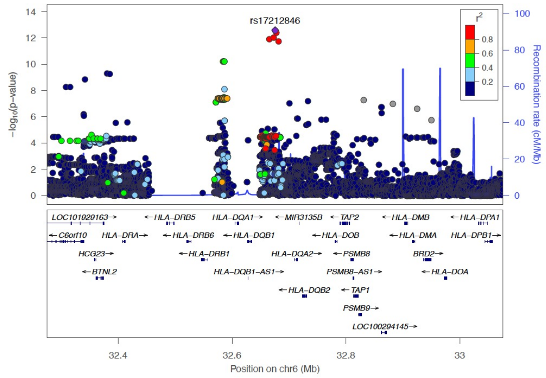

D. Conditional analysis on rs9271602, rs9391784, & rs171212846

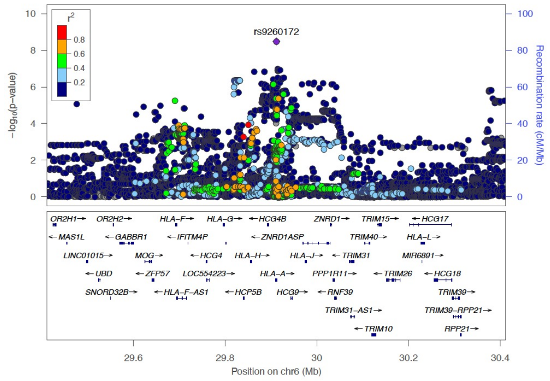

Supplementary Figure S3: Conditional analysis in HLA-DQ/DR region identified in Sri Lankan discovery cohort

A) LocusZoom plot for lead SNP rs9271602 before conditioning; B) Locus zoom after conditioning on rs9271602. The SNP rs9391784 has the lowest p-value after this conditioning and is upstream of *HLA-DRB5;* C) Locus zoom after conditioning on both rs9271602 and rs9391784, revealing a third independent signal at rs17212846 between *HLA-DQB1* and *HLA-DQA2;* D) Locus zoom after conditioning on rs9271602, rs9391784, and 17212846. A fourth signal at rs9260172 appears in *HLA-A*. SNPs surrounding the lead SNPs that are coloured in red, yellow, or green are in LD with the index SNP as depicted by the r^2 value in the legend.

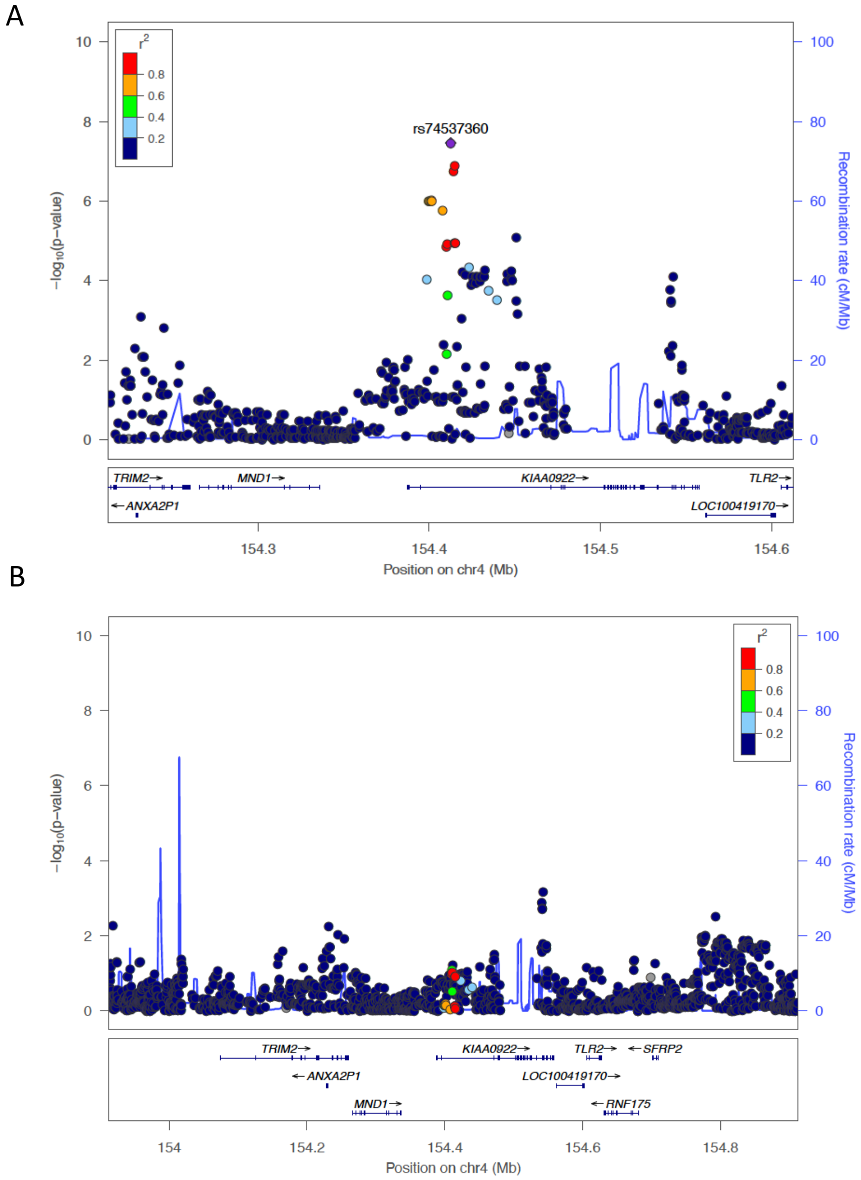

Supplementary Figure S4: Conditional analysis in 4q31.3 region (TMEM131L) identified in Sri Lankan discovery cohort

Each dot represents a SNP. Index SNPs are labelled as purple diamond. SNPs surrounding the lead SNPs that are coloured in red, yellow, or green are in linkage disequilibrium with the index SNP as depicted by the r^2 value in the legend. A) Locus zoom plot for lead SNP rs74537360 before conditioning; B) Locus zoom after conditioning on rs74537360, showing no further identified independent signals.

A

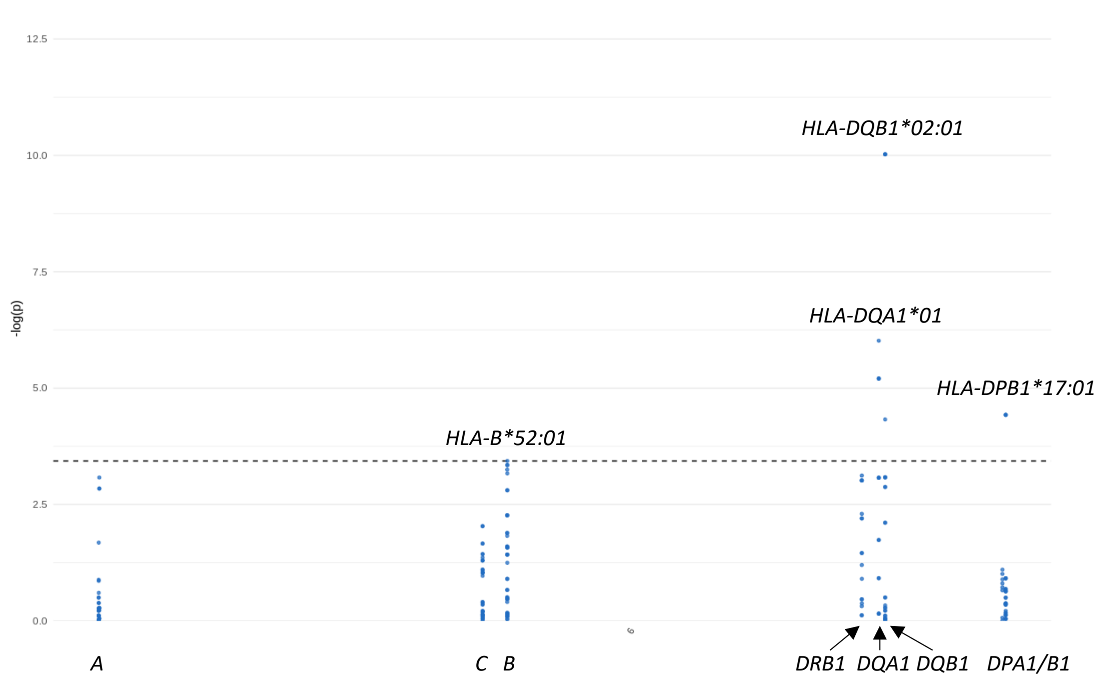

B

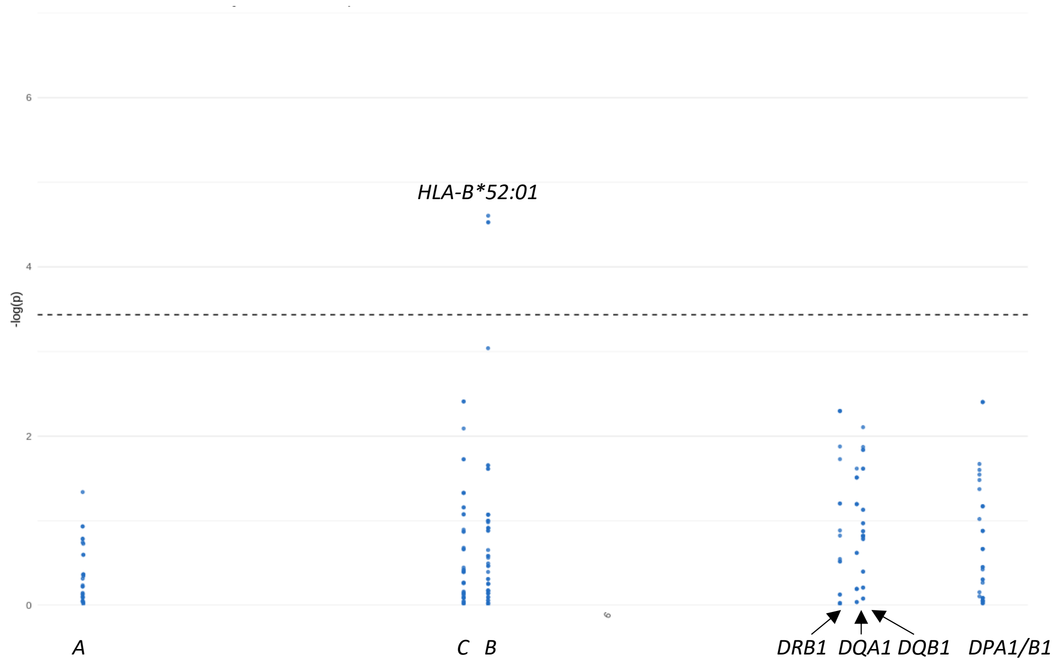

Supplementary Figure S5: HLA 4-digit allele analysis in the Sri Lankan discovery cohort

HLA genes (labelled) are listed along the x axis, with 4-digit subtypes listed over the top associations at each allele. The level of significance is depicted along the y axis as -$\log_{10}$(p). Each

dot represents a 4-digit HLA allele. The dashed line represents the threshold for Bonferroni-corrected statistical significance (p=0.05/136=0.00037).

A) Logistic regression analysis adjusted for the first ten principal components of ancestry on the imputed HLA dataset; B) conditional analysis on HLA-DQB1*02:01, showing an independent signal remained at HLA-B*52:01.

Supplementary Tables

SNP	Locus/gene	Test Allele	Meta-analysis (this study)					Sri Lankan (this study)		European (Dufek *et al(5)*)		Japanese (Jia *et al(22)*)	
			I2	P Heterogeneity	OR	95% CI	p-value	OR	p-value	OR	p-value	OR	p-value
rs2856665	*HLA-DR/DQ*	G	0	5.58E-01	4.06	3.47-4.75	2.45E-68	3.84	1.00E-26	4.22	3.71E-42	-	-
rs2637681	*CALHM6*	G	93	2.67E-04	0.62	0.54-0.71	6.69E-13	0.86	1.99E-01	0.52	3.53E-17	-	-
rs2746432	*AHI1*	C	0	8.19E-01	1.37	1.22-1.52	2.79E-08	1.34	8.35E-04	1.38	6.40E-06	-	-
rs4696465*	*TMEM131L*	G	80	2.37E-02	1.63	1.23-2.16	6.72E-04	1.98	4.50E-05	0.95	8.52E-01	-	-
rs10518133	*PARM1*	A	93	2.52E-04	1.59	1.31-1.93	2.95E-06	0.85	4.03E-01	1.96	2.50E-08	-	-
rs6478109	*TNFSF15*	A	0	8.17E-01	0.85	0.75-0.96	9.96E-03	0.83	8.38E-02	0.86	4.89E-02	0.72	2.54E-08

Supplementary Table S1: Genome-wide significant variants associated with SSNS published in European, Japanese, and Sri Lankan populations

*This is a proxy SNP for the lead variant (rs7437360) at *TMEM131L* in the Sri Lankan discovery GWAS (R^2=0.87), because the lead variant was not includ
the meta-analysis.

Allele frequencies for each SNP tabulated in column 1 are reported for European, Sri Lankan, and Japanese populations using data from Ensembl(23). Japan
lack of publicly available data from this study.

SNP, single nucleotide polymorphism; I^2, percentage of total variation across studies due to heterogeneity; P_heterogeneity, p-value of Cochrane Q test for he
OR, odds ratio

HLA Allele	Sri Lankan (this study)		European (Dufek et al(5))		AF SL	AF EUR	AF JAP
	OR	p-value	OR	p-value			
DQB1*02:01	2.24	2.59E-11	2.43	9.77E-22	0.17	0.21[+]	0.01
DQA1*01	0.59	5.92E-07	0.36	1.90E-31	0.52	0.36[+]	0.07
DQA1*02:01	1.72	3.99E-06	3.42	1.06E-32	0.18	0.15[+]	0.04
DPB1*17:01	4.04	2.80E-05	-	-	0.01	0.009[++]	0.01
DQB1*05	0.57	3.90E-05	-	-	0.25	0.15[+++]	0.07
B*52:01	2.00	2.98E-04	-	-	0.08	0.03[+++]	0.11
DRB1*07:01	-	-	3.26	5.62E-31	Unknown	0.15[+]	0.004
DQA1*01:03	-	-	0.24	9.77E-22	0.22	0.09[+]	0.19
DRB1*13	-	-	0.31	2.41E-14	0.09	0.08[+]	0.005
DRB1*13:01	-	-	0.23	3.18E-14	0.05	0.08[+]	0.005
DQA1*01:01	-	-	0.46	1.53E-10	0.17	0.15[+]	0.06
B*08:01	-	-	2.95	9.17E-09	0.02	0.13[+]	0.0001

Supplementary Table S2: HLA alleles associated with SSNS in Sri Lankan and European populations
Note that there were no overlapping HLA alleles associated with SSNS in Japanese populations (Jia *et al*).
OR, Odds Ratio; AF, allele frequency; AF, allele frequency; SL, Sri Lankan; EUR, European; JAP, Japanese.
[+] values obtained from Dufek *et al(5)*; [++] values obtained from Lemin *et al(24)*; [+++] values obtained from Tokic *et al(25)*; all allele frequency values for the S[illegible] from the control cohort of the Sri Lankan discovery GWAS; all allele frequency values for the Japanese population were obtained from http://hla.or.jp

Supplementary References

S1. Cattran DC, Feehally J, Cook HT et al. Kidney disease: Improving global outcomes (KDIGO) glomerulonephritis work group. KDIGO clinical practice guideline for glomerulonephritis. Kidney International Supplements *[Internet]* 2012; [cited 2020 Apr 27] 2: 139–274. Available from: https://mayoclinic.pure.elsevier.com/en/publications/kidney-disease-improving-global-outcomes-kdigo-glomerulonephritis

S2. Webb NJA, Frew E, Brettell EA et al. Short course daily prednisolone therapy during an upper respiratory tract infection in children with relapsing steroid-sensitive nephrotic syndrome (PREDNOS 2): protocol for a randomised controlled trial. Trials 2014; 15: 147.

S3. Webb NJ, Woolley RL, Lambe T et al. Sixteen-week versus standard eight-week prednisolone therapy for childhood nephrotic syndrome: the PREDNOS RCT. Health Technology Assessment (Winchester, England) 2019; 23: 1–108.

S4. Noone DG, Iijima K, Parekh R. Idiopathic nephrotic syndrome in children. Lancet (London, England) 2018; 392: 61–74.

S5. Dufek S, Cheshire C, Levine AP et al. Genetic Identification of Two Novel Loci Associated with Steroid-Sensitive Nephrotic Syndrome. Journal of the American Society of Nephrology: JASN 2019; 30: 1375–1384.

S6. Sherry ST, Ward MH, Kholodov M et al. dbSNP: the NCBI database of genetic variation. Nucleic Acids Research 2001; 29: 308–311.

S7. Anderson CA, Pettersson FH, Clarke GM, Cardon LR, Morris AP, Zondervan KT. Data quality control in genetic case-control association studies. Nature Protocols 2010; 5: 1564–1573.

S8. Manichaikul A, Mychaleckyj JC, Rich SS, Daly K, Sale M, Chen W-M. Robust relationship inference in genome-wide association studies. Bioinformatics *[Internet]* 2010; [cited 2021 Oct 19] 26: 2867–2873. Available from: https://www.ncbi.nlm.nih.gov/pmc/articles/PMC3025716/

S9. Michigan Imputation Server [Internet]. [cited 2022 May 21] Available from: https://imputationserver.sph.umich.edu/index.html#!

S10. Auton A, Abecasis GR, Altshuler DM et al. A global reference for human genetic variation. Nature *[Internet]* 2015; [cited 2020 Sep 16] 526: 68–74. Available from: https://www.nature.com/articles/nature15393

S11. Zhou W, Nielsen JB, Fritsche LG et al. Efficiently controlling for case-control imbalance and sample relatedness in large-scale genetic association studies. Nature Genetics *[Internet]* 2018; [cited 2021 May 28] 50: 1335–1341. Available from: https://www.nature.com/articles/s41588-018-0184-y

S12. Pruim RJ, Welch RP, Sanna S et al. LocusZoom: regional visualization of genome-wide association scan results. Bioinformatics *[Internet]* 2010; [cited 2022 Mar 31] 26: 2336–2337. Available from: https://www.ncbi.nlm.nih.gov/pmc/articles/PMC2935401/

S13. Luo Y, Kanai M, Choi W et al. A high-resolution HLA reference panel capturing global [illegible]

S16. Dick DM, Nasim A, Edwards AC et al. Spit for Science: launching a longitudinal study of genetic and environmental influences on substance use and emotional health at a large US university. Frontiers in Genetics 2014; 5: 47.

S17. Sequencing of 53,831 diverse genomes from the NHLBI TOPMed Program | bioRxiv [Internet]. [cited 2020 Nov 13] Available from: https://www.biorxiv.org/content/10.1101/563866v1

S18. GTEx Consortium. The Genotype-Tissue Expression (GTEx) project. Nature Genetics 2013; 45: 580–585.

S19. Liao J, Yu Z, Chen Y et al. Single-cell RNA sequencing of human kidney. Scientific Data *[Internet]* 2020; [cited 2020 Sep 15] 7: 4. Available from: https://www.nature.com/articles/s41597-019-0351-8

S20. Liu H, Doke T, Guo D et al. Epigenomic and transcriptomic analyses define core cell types, genes and targetable mechanisms for kidney disease. Nature Genetics 2022; 54: 950–962.

S21. Lee CM, Barber GP, Casper J et al. UCSC Genome Browser enters 20th year. Nucleic Acids Research 2019;

S22. Jia X, Yamamura T, Gbadegesin R et al. Common risk variants in NPHS1 and TNFSF15 are associated with childhood steroid-sensitive nephrotic syndrome. Kidney International 2020; 98: 1308–1322.

S23. Yates AD, Achuthan P, Akanni W et al. Ensembl 2020. Nucleic Acids Research *[Internet]* 2020; [cited 2022 Aug 31] 48: D682–D688. Available from: https://www.ncbi.nlm.nih.gov/pmc/articles/PMC7145704/

S24. Lemin AJ, Foster L. HLA-DPB1 allele frequencies in the West Midlands region of the United Kingdom: A critical evaluation against the common, intermediate and well-documented allele catalogues CWD 2.0.0, EFI CWD and CIWD 3.0.0. HLA *[Internet]* 2021; [cited 2022 Aug 1] 98: 5–13. Available from: http://onlinelibrary.wiley.com/doi/abs/10.1111/tan.14291

S25. Tokić S, Žižkova V, Štefanić M et al. HLA-A, -B, -C, -DRB1, -DQA1, and -DQB1 allele and haplotype frequencies defined by next generation sequencing in a population of East Croatia blood donors. Scientific Reports *[Internet]* 2020; [cited 2022 Aug 1] 10: 5513. Available from: https://www.nature.com/articles/s41598-020-62175-9
